# DNA damage response and cancer therapeutics through the lens of the Fanconi Anemia DNA repair pathway

**DOI:** 10.1186/s12964-017-0195-9

**Published:** 2017-10-10

**Authors:** Sonali Bhattacharjee, Saikat Nandi

**Affiliations:** Cold Spring Harbor Laboratory, New York, USA

**Keywords:** DNA repair, Fanconi Anemia (FA) signaling network, DNA damage response, Cancer therapeutics, Synthetic lethality, Combination Therapy Genomic instability, Interstrand crosslink (ICL), Homologous recombination, Translesion synthesis

## Abstract

Fanconi Anemia (FA) is a rare, inherited genomic instability disorder, caused by mutations in genes involved in the repair of interstrand DNA crosslinks (ICLs). The FA signaling network contains a unique nuclear protein complex that mediates the monoubiquitylation of the FANCD2 and FANCI heterodimer, and coordinates activities of the downstream DNA repair pathway including nucleotide excision repair, translesion synthesis, and homologous recombination. FA proteins act at different steps of ICL repair in sensing, recognition and processing of DNA lesions. The multi-protein network is tightly regulated by complex mechanisms, such as ubiquitination, phosphorylation, and degradation signals that are critical for the maintenance of genome integrity and suppressing tumorigenesis. Here, we discuss recent advances in our understanding of how the FA proteins participate in ICL repair and regulation of the FA signaling network that assures the safeguard of the genome. We further discuss the potential application of designing small molecule inhibitors that inhibit the FA pathway and are synthetic lethal with DNA repair enzymes that can be used for cancer therapeutics.

## Background

Fanconi Anemia (FA), a rare genetic cancer-susceptibility syndrome is a recessive autosomal or X-linked genetic disease [1–3]. FA is characterized by genomic instability, bone marrow failure leading to progressive aplastic anemia, chromosomal fragility and heightened susceptibility to cancer, particularly acute myelogenous leukemia (AML) [1, 4]. With an incidence of ~1–5 per 1,000,000 births, many FA patients suffer from developmental disorders and physical abnormalities ranging from short stature, abnormal skin pigmentation, organ malformation, hypogonadism, and developmental delay [5]. Patients are often diagnosed with early onset of solid tumors including squamous cell carcinomas of the head and neck, cervical cancer and liver tumors [6, 7]. FA was first described by the Swiss pediatrician Guido Fanconi in 1927 while treating a family of five siblings, three of whom presented with developmental birth defects and died from an early-onset of clinical features resembling pernicious anemia [8]. Additional clinical features included microcephaly, vitiligo and hypoplasia of the testes [8]. After nearly four decades another article reported an accumulation of large number of chromatid breaks in the blood lymphocytes of FA patients [9]. Due to high frequencies of chromosomal abnormalities, predominantly chromatid breaks during S-phase of the cell cycle, researchers concluded that FA patients have impaired double strand break repair (DSBR) [10]. Also despite the varied clinical phenotypes of the disease, a defining characteristic of FA cells is the cellular hypersensitivity to DNA crosslinking agents such as mitomycin C (MMC), chemotherapeutic agent cisplatin (CDDP), and diepoxybutane (DEB) [9, 11–15]. These crosslinks block ongoing DNA replication, DNA transcription, and if left unrepaired, activate cell apoptosis [16]. The observation that a functional FA pathway is required for processing damage after exposure to crosslinking agents has led to a great deal of research implicating the FA pathway in crosslink repair and the maintenance of genomic stability [17, 18]. Additionally, since the FA pathway has also been associated with cancer susceptibility, a better understanding of the mechanisms and roles of this pathway will enable the development of better-targeted cancer therapeutics.

In this review will we will focus on the repair of DNA interstrand crosslinks (ICLs) by the FA network of proteins. We aim to summarize our current understanding of ICL repair largely based on studies in the mammalian system. We will discuss the etiology of ICLs, the DNA repair pathways involved in the repair of ICLs, FA proteins, FA-DNA repair network and conclude with a perspective on targeting the FA pathway to identify anticancer therapeutic strategies.

### Interstrand crosslinks

ICLs are highly toxic DNA lesions that prevent the separation of the Watson and Crick strands of the double helix by covalently linking the two DNA strands. In doing so ICLs block critical cellular processes such as transcription and replication. ICLs can lead to gross-chromosomal aberrations like chromosome deletion, chromosome loss and DNA breaks [19]. The ability of ICLs to impede DNA replication and thereby block cell proliferation is used in chemotherapy to treat various cancers [20]. Chemotherapeutic drugs like cisplatin and its derivatives, carboplatin and oxaliplatin are bifunctional alkylating agents that form ICLs [21]. Although ICL repair remains poorly understood, factors involved in nucleotide excision repair (NER), homologous recombination (HR), and translesion synthesis (TLS) have been implicated in ICL removal and subsequent repair [22]. In non-proliferating cells such as quiescent cells, NER plays an important role in ICL recognition and removal [23, 24]. In contrast, in cells undergoing genome duplication, the DNA replication machinery serves as a sensor for ICLs. This subsequently triggers DNA damage checkpoint activation and initiates repair. In these S-phase cells, HR and TLS are the DSBR pathways employed for ICL repair [24]. In the past several years the role of FA network of proteins in the detection and repair of ICLs by promoting HR has been much better understood.

### Mechanistic insights into replication-dependent ICL repair

**Fig. 1:**
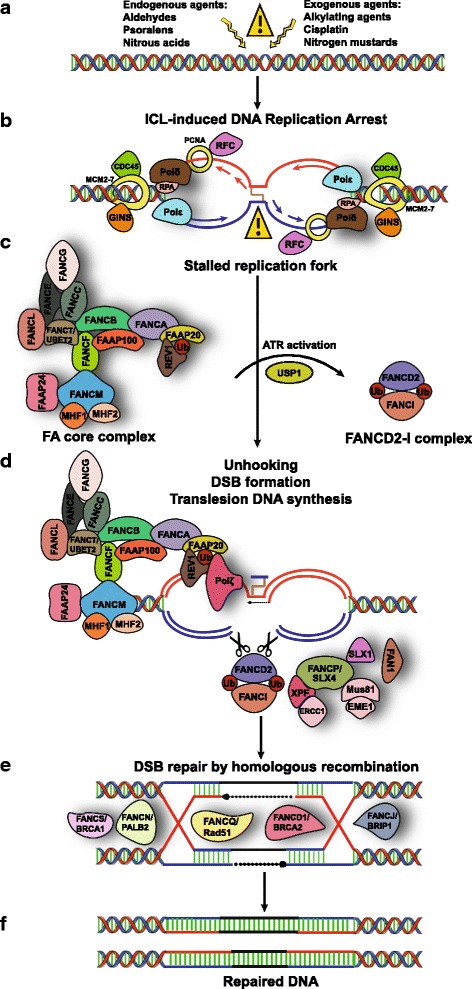
A model for the DNA interstrand crosslink (ICL) repair: Crosstalk between the Fanconi Anemia (FA) pathway, translesion synthesis (TLS) and homologous recombination (HR). **a** Certain endogenous, environmental sources and chemotherapeutic agents inflict damage to the DNA forming adducts between each DNA strands creating inter-strand crosslinks. **b** Two replication forks converge at the DNA ICL covalently linking the Watson and Crick strands of the DNA. The replication machinery encounters the DNA lesion at the fork leading to fork stalling. **c** The FA core complex detects the stalled replication fork, assembles on the DNA lesion and initiates checkpoint response by activating ATR, which in turn phosphorylates multiple FA proteins. This triggers the ubiquitin ligase activity of FANCL resulting in monoubiquitination of FANCD2 and FANCI. **d** The FANCD2-FANCI heterodimeric complex is recruited to the ICL site. This further recruits downstream nucleases, in particular structure specific endonucleases like SLX4 (FANCP), ERCC1-XPF, FAN1 and MUS81-EME1 to coordinate nucleolytic incisions flanking the ICL. The incisions unhook the ICL leaving crosslinked nucleotides tethered to the complementary strand. FAAP20 interacts with the FA core complex and binds to monoubiquitinated REV1. This catalyze TLS-dependent lesion bypass across the adduct, mediated by specialized TLS polymerases such as REV1 and Polζ. This restores the integrity of the template strand required for the progression of the nascent leading strand. **e** DSB generated after nucleolytic incisions serves as a suitable substrate for repair by the HR pathway. Downstream FA proteins promote RAD51-dependent strand invasion forming the synaptic filament. Branch migration and intermediates containing Holliday junctions are formed. **f** The resulting double Holliday junction is resolved by HR specific nucleases, HR repair is completed and the integrity of the DNA is restored

ICL repair is initiated when a traveling replication fork is stalled due to collision with a lesion on the DNA that triggers the activation of the DNA repair machinery [12, 22, 25]. Structure-specific endonucleases generate incisions on either side of the ICL, followed by TLS and then HR-mediated replication fork restart allows for the rescue of such stalled forks [12] (Fig. 1). It is important to note that majority of ICL repair in dividing cells is coupled to DNA replication. In mammalian cells, irrespective of the cell-cycle phase where the ICL is formed, the repair occurs exclusively during S-phase i.e., replication-dependent ICL repair [26].

Mechanistic details of replication-dependent ICL repair emerged from studies in *Xenopus* egg extracts where replication-coupled ICL repair was reconstituted in vitro by using site-specific ICL templates [27]. When a plasmid containing a site-specific ICL is incubated in this cell-free system, replication initiates at multiple origins of replication sites on the plasmid with two replication forks converging on the ICL. Initially, the leading strand polymerases stall ~20 nucleotides from the crosslink due to steric hindrance by the replisome (replicative helicase complex consisting of Cdc45, MCM2-7 and the GINS, collectively referred to as the CMG complex, and the replication polymerase) [27–29] which travels along the leading strand template and pauses at the lesion [30] (Fig. 1). After the initial fork pause, the stalled CMGs are unloaded and lesion bypass is initiated when the leading strand of a single fork is extended to within 1 nucleotide of the ICL lesion [30, 31]. Concurrent with this, the structure-specific endonucleases localize to the site of the ICL and promote dual incisions on either side of the ICL, a process also referred to as “unhooking” of the ICL [32]. A number of endonucleases have been implicated in the incision events of ICL repair including the 3′ flap endonuclease XPF-ERCC1, MUS81-EME1, FAN1, the 5′ flap endonuclease SLX1 and the scaffolding protein SLX4 [33–44]. TLS polymerases then fill in the gap at the site of the DNA incision. TLS incorporates a nucleotide across the ICL lesion by utilizing the error-prone DNA polymerase ζ. This allows the leading strand to be extended and ligated to the first downstream Okazaki fragment [12, 45, 46]. Finally, the broken sister chromatids generated by incision generates a DSB in the DNA that is repaired by RAD51-mediated HR utilizing the intact sister chromatid as a homology donor [47, 48] (Fig. 1).

In recent years the role of FA network of proteins in replication-dependent ICL repair has been the subject of intense research in many laboratories. In this section, we summarize the functions of the FA network of proteins in ICL repair and discuss the mechanisms by which they function in the repair of ICLs by promoting HR.

### Overview of the Fanconi Anemia DNA damage response pathway

The FA pathway is a nuclear multi-protein network comprised of 20 complementation groups and associated genes. Interestingly, 19 of the 20 genes of this network are autosomally inherited with the notable exception of FANCB. FANCB is localized on the X chromosome and its mutation has only been observed in males [2]. The genes were identified by methods such as, complementation analysis of cell lines from different FA patients, positional cloning, biochemical purification, and by sequencing candidate genes [49, 50]. The proteins encoded by these genes make up the FA network of proteins that cooperate in the DNA damage response (DDR) for the cellular resistance to ICLs (Fig. 1). These proteins have been placed into three groups based on the stage of ICL repair they participate in [15]. Group I, also referred to as the FA core complex consists of FANCA, FANCB, FANCC, FANCE, FANCF, FANCG, FANCL, FANCM and FANCT (UBET2) along with five additional proteins that associate with the FA core complex, including FAAP100, FAAP24, FAAP20, and the histone fold dimer proteins MHF1 and MHF2 [51–61]. Group II also referred to as the ID complex consists of FANCD2 and FANCI proteins [62–64]. Group III proteins include the DNA repair factors including HR proteins BRCA2 (FANCD1), BRIP1 (FANCJ), PALB2 (FANCN), RAD51C (FANCO), RAD51 (FANCR), SLX4 (FANCP), BRCA1 (FANCS), and XRCC2 (FANCU), TLS gene REV7 (FANCV) and DNA endonuclease XPF (FANCQ) [60, 65, 66]. Some patients with FA-like cellular phenotypes are yet to be assigned a FA-subtype indicating that additional FA or FA-associated genes are yet to be identified [11].

### The FA Core complex

FANCM is a DNA translocase which together with Fanconi anemia-associated protein 24 (FAAP24), FAAP 100 and the histone fold proteins MHF1 (FAAP16 or CENPS) and MHF2 (FAAP10 or CENPX) is responsible for lesion recognition and recruitment of the core complex which comprises of FANCA, FANCB, FANCC, FANCE, FANCF, FANCG, FANCL, FANCT, and FAAP20 to the ICL site [56, 67–69] (Fig. 1). It is important to note that recruitment of FANCM to ICLs is dependent on its phosphorylation by the ataxia telangiectasia and RAD3-related (ATR) checkpoint kinase [70]. Once recruited to the site of damage, the FA core complex serves as a multi-subunit ubiquitin E3 ligase for two other FA proteins, FANCD2 and FANCI [71]. FANCD2 is phosphorylated in an ATR-dependent manner which is essential for FANCD2 monoubiquitination and the establishment of the intra-S-phase checkpoint response [72]. Phosphorylation of FANCI is also essential for the monoubiquitination and localization of the FANCD2–I heterodimeric complex to DNA damage sites [73]. The phosphorylated FANCD2–I complex is subsequently monoubiquitinated by the FA core complex through its catalytic subunits, FANCL (the E3 ligase) and UBE2T (the ubiquitin E2 ligase also known as FANCT) [74–77]. Ubiquitinated PCNA also stimulates FANCD2 and FANCI monoubiquitination in vitro [78–80]. The ubiquitinated FANCD2–I complex is then recruited to chromatin by UHRF1 (ubiquitin-like with PHD and RING finger domains 1) protein that is involved in ICL sensing [81, 82].

Ubiquitination of FANCD2–I is a reversible regulatory modification. Deubiquitination of the FANCD2–I complex is required to release FANCD2 from the DNA repair complex crucial for subsequent repair steps to complete ICL repair [83–85]. The deubiquitination of FANCD2–I relies on USP1 (ubiquitin carboxy-terminal hydrolase 1) in conjunction with UAF1 (USP1-associated factor 1) [83, 86].

### DNA incision and Translesion repair

Ubiquination of the FANCD2–I complex is crucial for the recruitment of nucleases to the site of the ICL to orchestrate nucleolytic incision of the ICL. This facilitates ‘unhooking’ of the ICL from one of the two parental DNA strands to uncouple one sister chromatid from the other [32] (Fig. 1). FANCD2-Ub recruits the nuclease scaffold protein SLX4 (FANCP) by an interaction with ubiquitin-recognizing UBZ4 motif [35, 36]. SLX4 (FANCP) functions as a molecular platform to coordinate, recruit and activate other structure-specific endonucleases like XPF-ERCC1, MUS81-EME1 and SLX1 to aid ICL repair [87–90]. Interestingly, in vitro studies have shown that XPF–ERCC1–SLX4 complex is the essential nuclease for ICL unhooking whereas MUS81-EME1, SLX1 and FAN1 (Fanconi-associated nuclease 1, another structure-specific nuclease that acts in a FANCP independent manner) possess redundant ICL processing activities [44, 91]. It is important to note that in human cells, the recruitment of XPF at sites of ICL damage is dependent on the structural protein non­erythroid α­spectrin (αIISp) during the S-phase of the cell cycle [92–94]. After unhooking of the ICL lesion, ubiquitinated PCNA and the FA core complex recruit translesion synthesis polymerases to coordinate the next step of ICL repair. Translesion DNA polymerases such as REV7 (FANCV), polymerase ζ and polymerase η fill the single-strand DNA (ssDNA) gaps resulting from ICL unhooking. Translesion DNA polymerases have larger binding pockets compared to replicative polymerases and can accommodate bulky ICL adducts thereby incorporating nucleotides opposite to the ICL and filling the DNA gap [95, 96].

### Downstream Effector complex

In addition to ssDNA gaps formed in one strand of the double helix, unhooking results in the formation of DSB afflicting both strands. Repair of DSBs relies on the HR pathway (Fig. 1). Consistent with this, cells deficient in HR proteins display hypersensitivity to ICL agents [47, 97]. FA proteins involved in HR are not required for FANCD2–I monoubiquitination suggesting they function downstream of the FANCD2–I complex. Several FA factors have been shown to promote different stages of HR [60]. BRCA2 (FANCD1), FANCO (RAD51C) and PALB2 (FANCN) help load RAD51 onto ssDNA by displacing RPA, which specifically promotes RAD51-dependent nucleofilament formation and also stimulates RAD51-dependent strand invasion of a homologous DNA template [98–100]. End resection is a key step in DSBR and initiates HR. FANCD2 and BRCA1 (FANCS) promote the recruitment of the resection factor CtIP at the site of DSBs to initiate HR [101–104]. FANCC has been implicated in inhibiting non-homologous end joining (NHEJ) factors from accessing the DSB ends thus preventing NHEJ and thereby promoting HR [105]. FANCJ’s (BRIP) 5′ to 3′ helicase activity has been shown to unwind D-loops and may be involved in resolving RAD51 nucleofilaments [106].

### Regulation of the FA network of proteins

ICL repair is a highly complex process involving the FA pathway as well as other repair pathways that needs to be tightly controlled. Post-translational modifications (PTMs) and protein-protein interactions are crucial for the regulation of this process. ATR plays a major regulatory role in the activation of the FA pathway. This kinase is responsible for the phosphorylation of the FANCD2-I heterodimer in the S-phase, which is indispensible for efficient FANCD2 ubiquitination and focus formation [72, 107, 108]. ATR also phosphorylates FANCA, FANCG and FANCM to promote efficient crosslink repair [109–113]. Chk1 also negatively regulates the FA pathway by phosphorylating FANCE to trigger its proteasomal degradation [114]. Ubiquitination of various FANC proteins is crucial for the regulation of the FA pathway. Monoubiquitination of the FANCD2-I complex by the FANCL-UBE2T is crucial for recruitment of the core complex to damaged DNA [115, 116]. Additionally, ubiquitination of effector proteins like FANCN, FANCS and FANCG have been implicated in the regulation of ICL repair [117, 118]. Deubiquitination of FANCD2 and FANCI by the constitutively active deubiquitinating complex UAF1-USP1 keeps the pathway turned off unless required [86]. Upon DNA damage, the activity of UAF1-USP1 is repressed either by proteosomal degradation of USP1 or by transcription repression of the USP1 gene [86]. Finally, SUMOylation plays a pivotal role in the regulation to FA-mediated ICL repair [119]. SUMOylation of FANCD2 and FANCI by PIAS1/4 and UBC9 promotes polyubiquitination of the complex, which in turn promotes dissociation of FANCD2 and FANCI from chromatin [120].

### FA factors as therapeutic targets in cancer

A hallmark of cancer cells is genome instability. This can be attributed to a failure of the DNA repair machinery, which essentially acts as a tumor suppressor network to preserve genome integrity and prevent malignancy. The link between FA and cancer predisposition has been well established with FA patient populations exhibiting a wide range of cancers [121]. Almost 25% of FA patients develop malignancies [121]. Although the most common malignancies are either hematologic, like myelodysplastic syndrome and AML or solid tumors, particularly squamous cell carcinomas of the head and neck [121], recently FA proteins mutations have been reported in familial and sporadic cancers outside the FA patient population [121]. For instance, FANCD1 mutations have been associated with ovarian, breast, prostate, stomach and pancreatic cancers [122]. FANCL mutations have been associated with lung cancer, pancreatic cancer, breast cancer and leukemia [123, 124]. FANCD2 mutations have been associated with breast cancer [125]. FANCN mutations have been reported in prostate and breast cancer [126]. FANCC and FANCG have also been implicated in pancreatic cancer, breast cancer and leukemia [124, 127, 128].

### Leveraging synthetic lethal interactions with the FA pathway for cancer therapeutics

A major drawback of chemotherapy lies in the fact that it is not selective, i.e., it kills both cancer cells and normal cells indiscriminately. However, inactivation/defects in DNA repair pathways can make cancer cells over-dependent on a compensatory DNA repair pathway for survival. Current approaches for cancer therapy that rely on inhibiting the intact functional DNA repair pathways by using a synthetic lethal approach can provide a therapeutic strategy for specific killing of such tumors. Two genes are said to be in a synthetic lethal relationship if a mutation in either gene alone is not lethal but simultaneous mutations are lethal [48, 129]. A new approach is directed at exploiting the synthetic lethality of cancer cells that are defective in the FA pathway [130].

**Fig. 2:**
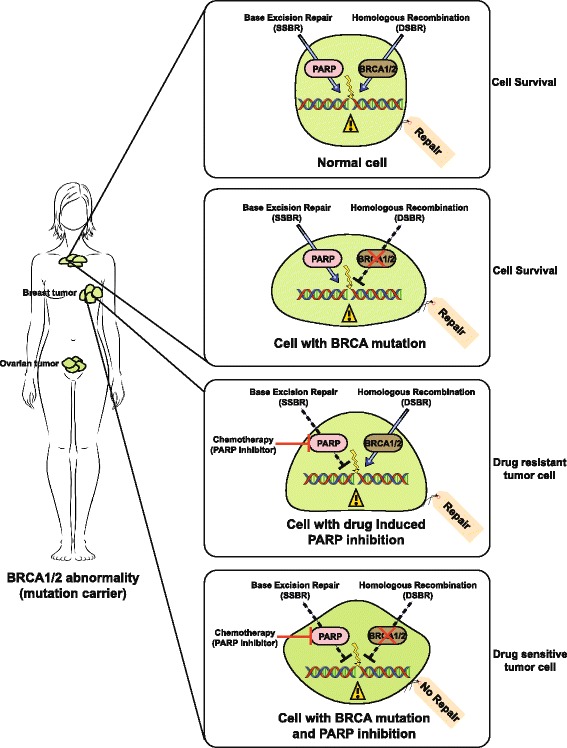
Synthetic lethal interactions to identify molecular targets for cancer therapy: Sensitizing genetically defined tumor cells by targeted inhibition of DNA damage repair pathways. A model for synthetic lethality using PARP inhibitors. In breast/ovarian tumor cells, mutation in BRCA1/2 leaves the cancer cell vulnerable to chemotherapeutic drugs against single strand break repair (SSBR). In contrast, cells with functional BRCA1/2 genes are spared as they can repair the lesions on the DNA using double strand break repair (DSBR) pathway. Compromised base excision repair (BER) pathway combined with homologous recombination (HR) deficiency leads to tumor cell death

The best example of the therapeutic potential of the synthetic lethality approach is development of poly(adenosine diphosphate [ADP]–ribose) polymerase 1 (PARP1) inhibitors to treat breast and ovarian cancers carrying mutations in the tumor-suppressor genes BRCA1 or BRCA2 [131, 132] (Fig. 2). Recognition of DNA breaks by PARP1 is one of the earliest events in DSBR. Once a DNA strand break is formed, PARP1 binds to the broken DNA ends and facilitates chromatin decondensation at the break site [133]. This allows repair enzymes to access the damaged DNA sites [133]. Inhibition or deletion of PARP1 leads to inactivation of the single strand break repair (SSBR) pathways including NER, base excision repair (BER), mismatch repair (MMR) which leads to the accumulation SSBs which may subsequently lead to the formation of DSBs [133]. BRCA1 and BRCA2 are also key participants in HR. In normal cells, loss of activity of PARP1 enzyme induces high levels of DSBR through the HR pathway during the S-phase of the cell cycle. Cancer cells that are defective in HR are selectively sensitive to PARP inhibition due to the simultaneous loss of two DNA repair pathways. Thus, treating cells carrying BRCA1 or BRCA2 mutations with small-molecule inhibitors of PARP1 are lethal as the cells are deficient in DSBR. This results in targeted killing of the cancerous cells, while cells with intact HR can repair the damage and survive [134] (Fig. 2).

Synthetic lethal interactions with the FA pathway for the development of inhibitors have been explored. A siRNA-based synthetic lethal screening identified several genes including ATM, PARP1, CDK1, NBS1, and PLK1 that showed synthetic lethal interactions with FANCG, indicating that these genes could be targeted concomitant with a FA pathway inhibitor [135]. Since ATM deficiency has been reported in triple- negative breast cancer and several types of hematological malignancies like mantle cell lymphoma, chronic lymphocytic leukemia, and acute lymphoblastic leukemia [136, 137], the FA pathway inhibitor could have immense therapeutic potential. CHK1 inhibition has also been shown to be synthetically lethal with FANCA deficiency following cisplatin treatment [138].

Several small molecule inhibitors have been identified that inhibit specific components of the FA pathway. This in turn leads to inhibition of FANCD2 foci formation and abrogation of the FA pathway. For example, wortmannin (inhibits ATR kinase), H-9 (inhibits several kinases including protein kinase A, G, and C), alsterpaullone (inhibits cyclin-dependent kinase 1 and 5), phenylbutyrate (inhibits FANCS) and curcumin (inhibits FANCF) are some of the small-molecule inhibitors of the FA/BRCA pathway that have already been identified by high-throughput screen using human cells and are now in various stages of subsequent validation [139, 140]. Bortezomib, the natural compound curcumin and its analogs such as EF24 and 4H-TTD and MLN4924 have been shown to impair FANCD2 activation and sensitize cancer cells to ICL-inducing agents [18, 139, 141]. USP1 inhibitors like C527, pimozide and GW7647 affect the ubiquitin-deubiquitination cycle of FANCD2 leading to the selective inhibition of the FA pathway [142–144]. Understanding the mechanism by which these compounds chemically inhibit the FA/BRCA2 pathway is crucial for translating this research from the laboratory to the clinic. For instance, phenylbutyrate sensitizes head and neck cancer cells to cisplatin by specifically attenuating FANCS thereby inhibiting FANCD2 foci formation and abrogating the FA/BRCA pathway [140]. This observation makes phenylbutyrate an excellent candidate for sensitizing cisplatin-resistant head and neck tumors in a clinical setting [140]. Curcumin (diferuloylmethane), a low-molecular-weight polyphenol and a component in the spice turmeric inhibits FANCF [139]. Since FANCF acts upstream in the FA/BRCA pathway, inhibition of FANCF attenuates monoubiquitination of FANCD2 and FANCD2 foci formation [139]. In ovarian and breast tumor cell lines, curcumin-mediated inhibition of the FA/BRCA pathway sensitizes tumor cells to cisplatin by inducing apoptotic cell death. This opens up the possibility that curcumin could be used to sensitize cisplatin-resistant ovarian and breast tumors in the clinic. The precise inhibition of the FA pathway in combination with DNA repair inhibitors could increase the efficacy of chemotherapy and improve current cancer treatment regimens.

## Conclusion

Understanding the molecular details of the DNA damage response is essential for advancing cancer research. Due to the critical importance of the FA network in maintaining genome stability and the current limitations in treating FA patients in the clinic, a large body of research has been directed to this subject. The FA pathway plays a central role in ICL repair during which the FA proteins function to coordinate NER factors, TLS polymerase, HR factors and checkpoint kinases to ensure genome stability. In the absence of a functional FA pathway, cells are predisposed to spontaneous and DNA damage-induced chromosomal breaks. More research into the FA DNA repair pathway will identify novel factors that can be specifically inhibited. Such targeted modulation of the FA pathway by exploiting synthetic lethal relationships may play an important role for the development of new cancer treatments and potential development of personalized therapies.

## Abbreviations

AML: Acute myelogenous leukemia
ATR: Ataxia telangiectasia and RAD3-related
CDDP: Chemotherapeutic agent cisplatin
DDR: DNA damage response
DEB: Diepoxybutane
DSB: Double strand break
DSBR: Double strand break repair
dsDNA: Double-strand DNA
FA: Fanconi Anemia
FAN1: Fanconi-associated nuclease 1
HR: Homologous recombination
ICLs: Interstrand DNA crosslinks
MMC: Mitomycin C
NER: Nucleotide excision repair
PTMs: Post-translational modifications
ssDNA: Single-strand DNA
TLS: Translesion synthesis
UAF1: USP1-associated factor 1
UHRF1: Ubiquitin-like with PHD and RING finger domains 1
USP1: Ubiquitin carboxy-terminal hydrolase 1
